# A Study of Molecular Mimicry and Immunological Cross-reactivity between Hepatitis B Surface Antigen and Myelin Mimics

**DOI:** 10.1080/17402520500285247

**Published:** 2005-09

**Authors:** Dimitrios-Petrou Bogdanos, Heather Smith, Yun Ma, Harold Baum, Giorgina Mieli-Vergani, Diego Vergani

**Affiliations:** 1 Institute of Liver Studies King's College London School of Medicine at King's College Hospital Denmark Hill London SE5 9RS UK; 2 School of Biomedical and Health Sciences King's College London SE1 9NN UK

## Abstract

On the basis of the reported association between hepatitis B vaccination (HBvacc) and autoimmune demyelinating complications such as multiple sclerosis (MS), we have looked for aminoacid similarities between the small hepatitis B virus surface antigen (SHBsAg), and the MS-autoantigens myelin basic protein (MBP) and myelin oligodendrocyte glycoprotein (MOG) that could serve as targets of immunological cross-reactivity. Twenty-mer peptides spanning 4 SHBsAg/MOG and 1 SHBsAg/MBP mimicking pairs, were constructed and tested by ELISA as targets of cross-reactive responses. A total of 147 samples from 58 adults were collected before HBvacc (58/58), and post-HBvacc (48/58 before the second and 41/58 before the third boost). Eighty-seven sera from anti-SHBsAg antibody negative patients with various diseases were tested as pathological controls. Reactivity to at least one of the SHBsAg peptides was found in 8 (14%) pre-HBvacc subjects; amongst the remaining 50, reactivity to at least one of the SHBsAg peptides appeared in 47 (94%) post-HBvacc. Reactivity to at least one of the MOG mimics was present in 4 (8%) pre-HBvacc and in 30 (60%) post-HBvacc (*p* < 0.001). Overall 30/50 (60%) vaccinees had SHBsAg/MOG double reactivity on at least one occasion compared to none before-vaccination and in 2 (2%) of the pathological controls (*p* < 0.001 for both). SHBsAg/MOG double reactivity was cross-reactive as confirmed by inhibition studies. At 6 months post-vaccination, 3 of the 4 anti-MOG reactive cases before vaccination and 7 of the 24 (29%) of the anti-MOG reactive cases at 3 months post-vaccination had lost their reactivity to MOG_5-24_. There was no reactivity to the SHBsAg/MBP mimics. None of the vaccinees reported symptoms of demyelinating disorders. In view of the observed SHBsAg/MOG cross-reactivity, the vaccine's possible role as an immunomodulator of viral/self cross-reactivity must be further investigated.


*Clinical & Developmental Immunology*, September 2005; 12(3): 217–224

Taylor & Francis

Taylor & Francis Group

### A study of molecular mimicry and immunological cross-reactivity between hepatitis B surface antigen and myelin mimics

DIMITRIOS-PETROU BOGDANOS[1], HEATHER SMITH[1], YUN MA[1], HAROLD BAUM[2], GIORGINA MIELI-VERGANI[1], & DIEGO VERGANI[1]

[1]*Institute of Liver Studies, King's College London School of Medicine at King's College Hospital, Denmark Hill, London SE5 9RS, UK, and* [2]*School of Biomedical and Health Sciences, King's College, London SE1 9NN, UK*

**Abstract**
On the basis of the reported association between hepatitis B vaccination (HBvacc) and autoimmune demyelinating complications such as multiple sclerosis (MS), we have looked for aminoacid similarities between the small hepatitis B virus surface antigen (SHBsAg), and the MS-autoantigens myelin basic protein (MBP) and myelin oligodendrocyte glycoprotein (MOG) that could serve as targets of immunological cross-reactivity. Twenty-mer peptides spanning 4 SHBsAg/MOG and 1 SHBsAg/MBP mimicking pairs, were constructed and tested by ELISA as targets of cross-reactive responses. A total of 147 samples from 58 adults were collected before HBvacc (58/58), and post-HBvacc (48/58 before the second and 41/58 before the third boost). Eighty-seven sera from anti-SHBsAg antibody negative patients with various diseases were tested as pathological controls. Reactivity to at least one of the SHBsAg peptides was found in 8 (14%) pre-HBvacc subjects; amongst the remaining 50, reactivity to at least one of the SHBsAg peptides appeared in 47 (94%) post-HBvacc. Reactivity to at least one of the MOG mimics was present in 4 (8%) pre-HBvacc and in 30 (60%) post-HBvacc ($p < 0.001$). Overall 30/50 (60%) vaccinees had SHBsAg/MOG double reactivity on at least one occasion compared to none before-vaccination and in 2 (2%) of the pathological controls ($p < 0.001$ for both). SHBsAg/MOG double reactivity was cross-reactive as confirmed by inhibition studies. At 6 months post-vaccination, 3 of the 4 anti-MOG reactive cases before vaccination and 7 of the 24 (29%) of the anti-MOG reactive cases at 3 months post-vaccination had lost their reactivity to $MOG_{5-24}$. There was no reactivity to the SHBsAg/MBP mimics. None of the vaccinees reported symptoms of demyelinating disorders. In view of the observed SHBsAg/MOG cross-reactivity, the vaccine's possible role as an immunomodulator of viral/self cross-reactivity must be further investigated.

**Keywords:** *Autoantibody, autoimmunity, hepatitis B virus, multiple sclerosis, vaccination*

**Abbreviations:** *EAE, experimental autoimmune encephalomyelitis; SHBsAg, small hepatitis B surface antigen; MBP, myelin basic protein; MOG, myelin oligodendrocyte glycoprotein; MS, multiple sclerosis; OD, optical density; SI, stimulation index*

### Introduction

Molecular mimicry based on amino acid similarities shared by viral and self antigens has long been proposed as a pathogenic mechanism for autoimmune disease but documentation of this mechanism has been elusive in humans because infection probably occurs years before the clinically overt autoimmune disease (Dyrberg and Oldstone 1986; 1989; 1998; Bogdanos et al. 2000; Van de Water et al. 2001). Presently, proof for molecular mimicry relies on the availability of viral mimics able to induce cross-reactive immune responses leading to tissue damage in experimental animals such as experimental autoimmune encephalomyelitis (EAE), the animal model of multiple sclerosis and herpes stromal keratitis (Oldstone 1998; Wucherpfennig 2001; von Herrath et al. 2003).

The information derived from animal studies is of undoubted value but experimental models of human disease are not always able to reproduce faithfully

Correspondence: D. Vergani, Institute of Liver Studies, King's College London School of Medicine at King's College Hospital, Denmark Hill, London SE5 9RS, UK. Tel: +44 20 7346 3305. Fax: +44 20 7346 3700. E-mail: diego.vergani@kcl.ac.uk

ISSN 1740-2522 print/ISSN 1740-2530 online © 2005 Taylor & Francis
DOI: 10.1080/17402520500285247

the human condition. Hence, studies of immunological cross-reactivity in serum samples before and after vaccination against viruses may provide useful information as to how a viral stimulus can induce cross-reactive autoimmune responses. Indeed, it has been proposed that vaccination against infectious agents may activate pathways of molecular mimicry in genetically susceptible hosts, and this may be the basis of adverse reactions to vaccines (Cohen and Shoenfeld 1996; Poirriez 2004; Ravel et al. 2004). In the present study we have used vaccination against hepatitis B virus (HBVacc) as a model for the study of viral/self cross-reactivity.

The current HBVacc is a non-infectious viral subunit consisting of the small hepatitis B virus surface antigen (SHBsAg), a 226 aa long recombinant antigen identical to the "wild" SHBsAg (Shouval 2003). Extrahepatic autoimmune manifestations, such as demyelinating disorders and MS have increasingly been linked to HBVacc administration suggesting that the exposure to SHBsAg may be capable of inducing autoimmune adverse reactions (Herroelen et al. 1991; Cohen and Shoenfeld 1996; Maillefert et al. 1999; Shoenfeld and Aron-Maor 2000; Aron-Maor and Shoenfeld 2001; Vital et al. 2002; Poirriez 2004; Ravel et al. 2004). The fact that the HBVacc is administered in three doses over a period of seven months gives the opportunity to study the emergence of the humoral antiviral immune response and, at the same time, to witness appearance and evolution of the possible cross-reactive autoimmunity as a consequence of exposure to SHBsAg.

Among various myelin antigens, myelin basic protein (MBP) is the major target of MS and EAE (Raine and Bornstein 1970; Martin et al. 1992; Steinman 1996). More recent studies indicate that humoral immunity against myelin oligodendrocyte glycoprotein (MOG) is associated with myelin damage in MS (Devaux et al. 1997; Genain et al. 1999; Wekerle 1999). Having found through screening of protein databases that known epitopic regions on SHBsAg share extensive homologies with MBP and MOG, we have investigated whether the viral/self mimics serve as target of cross-reactive immune responses following HBVacc administration in healthy individuals.

### Material and methods

### *Subjects*

A total of 234 serum samples stored in the Institute of Liver Studies, King's College Hospital, London, were tested including 147 samples pre- and post-HB vaccination from 58 adults (median age 42, range 31–62, 38 male), negative for hepatitis B viral markers. Samples were collected before HBvacc (58/58), and post-HBvacc, at three months (48/58) before the second boost, and six months post-HBvacc (41/58) before the third boost. As pathological controls, 87 anti-HBsAg antibody negative patients (median age 45, range 22–74, 59 female) were tested including 23 with primary biliary cirrhosis (PBC), 27 with alcoholic liver disease (ALD), 19 with autoimmune thyroiditis, and 18 with systemic lupus erythematosus (SLE).

All sera were tested under code. Informed consent was obtained from each subject. The project was approved by the local ethical committee.

### *Anti-viral antibody detection*

A commercially available ELISA (Research Diagnostics, Flanders, NJ, USA) was used to detect IgG antibodies against SHBsAg.

### *Protein database search and analysis*

The "*BLASTp 2* sequences" programme (available at http://www.ncbi.nlm.nih.gov/ BLAST) was used to search for amino acid sequence similarity between SHBsAg and the MBP and MOG myelin antigens.

### *Peptide synthesis*

To assess cross-reactivity between SHBsAg and homologous self-sequences (Figure 1), seven 20-mer peptidyl mimics were constructed (Mimotopes Ltd, Clayton, Australia) as reported in Table I. An irrelevant, randomly generated 20-mer peptide (-HEDYVNQSLRPTPLEISVRA-) served as control (Bogdanos et al. 2002, 2004a; b, 2005; Kerkar et al. 2003). Peptides conformed to the following format: Biotin- SGSG-peptide-amide, in which SGSG is a spacer with alternating serine (S) and glycine (G) residues to avoid steric hindrance and reduce hydrophobicity. Biotin-captured peptides allow maximum absorbance at minimal antigen concentration, therefore providing a significant advantage over uncaptured peptides relying on random binding on plates, which at times requires high antigen concentrations and results in high background noise (Bogdanos et al. 2002; 2004a; b; 2005; Kerkar et al. 2003). Peptide purity was $\geq 90\%$ as determined by analytical reverse phase high-performance liquid chromatography. The identity of each purified peptide was confirmed by mass spectrometry. On arrival, 1 mg of each freeze-dried peptide was dissolved in 40% acetonitrile (Sigma-Aldrich Biochemical, Dorset, UK), aliquoted, and stored at $-70$°C.

### *Anti-peptide antibody detection*

Antibody binding to the peptides was determined by ELISA, as described previously (Bogdanos et al. 2002; 2004a; b; 2005; Kerkar et al. 2003). Briefly, 100 μl of

| Antigens | aa position | | | | | | | | | | | | | | | Identities | Similarities |
|---|---|---|---|---|---|---|---|---|---|---|---|---|---|---|---|---|---|
| SHBsAg | (135-140) | P | S | C | C | C | T | | | | | | | | | | |
| | | P | S | C | | C | . | | | | | | | | | 4 | 5/6(82%) |
| MOG | (10-15) | P | S | C | L | C | S | | | | | | | | | | |
| MOG | (12-20) | | C | L | C | S | F | L | L | L | L | | | | | | |
| | | | C | L | | | | L | . | L | L | | | | | 5 | 6/9 (66%) |
| SHBsAg | (89-98) | L | C | L | I | F | L | L | V | L | L | | | | | | |
| | | L | C | | | | L | L | . | L | L | | | | | 6 | 7/10 (70%) |
| MOG | (13-22) | L | C | S | F | L | L | L | L | L | L | | | | | | |
| SHBsAg | (83-89) | F | L | F | I | L | L | L | | | | | | | | | |
| | | F | L | | L | L | L | L | | | | | | | | 6 | 6/7(86%) |
| MOG | (16-22) | F | L | L | L | L | L | L | | | | | | | | | |
| SHBsAg | (9-15) | L | G | P | L | L | V | L | | | | | | | | | |
| | | L | G | P | L | . | . | L | | | | | | | | 5 | 7/7 (100%) |
| MOG | (218-224) | L | G | P | L | V | A | L | | | | | | | | | |
| SHBsAg | (67-80) | P | I | C | P | G | Y | R | W | M | C | L | R | R | F | | |
| | | P | . | | P | G | | R | | | | . | R | | F | 6 | 8/14 (57%) |
| MBP | (82-95) | P | A | D | P | G | S | R | P | H | L | I | R | L | F | | |

Figure 1. Amino acid sequence homology between small Hepatitis B surface antigen (SHBsAg) and myelin antigens. Amino acids in standard single letter; Full stop (.), conservative substitution. MOG, myelin oligodendrocyte glycoprotein; MBP, myelin basic protein.

peptide diluted in phosphate buffer saline (PBS, Sigma-Aldrich) containing 0.1% bovine serum albumin (BSA, Sigma-Aldrich) and 0.1% sodium azide (final peptide concentration, 5 μg/ml) was added to 96-well streptavidin coated microplates (Mimotopes). The optimal concentrations of reagents at various steps of the immunoassay were determined in preliminary experiments by checkerboard titration. After 90-minute incubation and washing, plates were incubated with 100 μl/well of serum diluted in 2% BSA/PBS containing 0.1% sodium azide (final dilution, 1/100). Plates were washed, and 100 μl/well of horseradish peroxidase- conjugated rabbit anti-total human immunoglobulin (Dako Ltd, High Wycombe, Bucks, UK), diluted 1/4000 in 2% BSA/PBS, was added. The reaction was developed by the addition of 100 μl/well of *o*-phenylenediamine solution containing 0.4 mg/ml of 3% hydrogen peroxide in citrate phosphate buffer (Sigma-Aldrich) and terminated with 100 μl/well of 4N H2SO4. Absorbance (optical density, OD) was read in a microplate reader (MRX; Dynex Technologies, West Sussex, UK) at 490 nm. Each serum tested against experimental peptides was also tested against the control peptide. The final

Table I. Single and double reactivity to SHBsAg and myelin mimics in absolute numbers and percentages (%), in 50 vaccinees before and after vaccination.

| | Pre-HBVacc baseline n=50 | Post-HBVacc 3 months n=45 | Post-HBVacc 6 months n=39 |
|---|---|---|---|
| $SHBsAg_{130\text{-}149}$ | 0/50 (0%) | 41/45(91%) | 37/39 (95%) |
| $SHBsAg_{80\text{-}99}$ | 0/50 (0%) | 1/45 (2%) | 0/39 (0%) |
| $SHBsAg_{1\text{-}20}$ | 0/50 (0%) | 10/45 (22%) | 13/39 (33%) |
| $SHBsAg_{65\text{-}84}$ | 0/50 (0%) | 5/45 (11%) | 5/39 13(%) |
| SHBsAg (at least 1peptide) | 0/50 (0%) | 42/45 (93%) | 37/39 (39%) |
| $MOG_{5\text{-}24}$ | 4/50 (8%) | 24/45 (53%) | 14/39 (36%) |
| $MOG_{210\text{-}229}$ | 1/50 (2%) | 8/45 (17%) | 5/39 (13%) |
| MOG (at least 1peptide) | 4/50 (8%) | 24/45 (53%) | 16/39 (41%) |
| $MBP_{80\text{-}99}$ | 0/50 (0%) | 0/45 (0%) | 0/39 (0%) |
| $SHBsAg_{130\text{-}149}/MOG_{5\text{-}24}$ | 0/50 (0%) | 23/45 (51%) | 16/39 (41%) |
| $SHBsAg_{80\text{-}99}/MOG_{5\text{-}24}$ | 0/50 (0%) | 1/45 (2%) | 0/39 (0%) |
| $SHBsAg_{1\text{-}20}/MOG_{210\text{-}229}$ | 0/50 (0%) | 7/45 (16%) | 5/39 (13%) |
| SHBsAg (full protein) | 0/50 (0%) | 40/45 (89%) | 36/39 (92%) |
| SHBsAg/MOG (at least 1pair) | 0/50 (0%) | 21/45 (47%) | 16/39 (41%) |

HBVacc, hepatitis B vaccination; SHBsAg, small hepatitis B surface antigen; MOG, myelin oligodendrocyte glycoprotein; MBP, myelin basic protein.

absorbance value was calculated as the absorbance (OD) $^{\text{test peptide}}$/OD $^{\text{control peptide}}$. Reaction to a given peptide was considered positive when $OD^{test}/OD^{control\ peptide}$ was $\geq 2$; this cut off representing mean + 5 SD of 135 readings using serum (tested in triplicate) from 15 healthy subjects against the mimicking peptides. Expression of absorbance as ratios of $OD^{test}/OD^{control\ peptide}$ avoids false-positive results caused by nonspecific binding to unrelated peptides in hypergammaglobulinemic serum samples (Bogdanos et al. 2002; 2004a; b; 2005; Kerkar et al. 2003). All experiments were done in triplicate.

*Inhibition studies*

To investigate whether the simultaneous reactivity to $SHBsAg_{130-149}$ and $MOG_{5-24}$ was due to cross-reactivity, competition ELISA were performed as previously described (Bogdanos et al. 2002; 2004a; b; 2005; Kerkar et al. 2003), measuring residual anti-$SHBsAg_{130-14}$ antibody reactivity after incubation respectively with $SHBsAg_{130-14}$, $MOG_{5-24}$, recombinant SHBsAg antigen (Research Diagnostics), the irrelevant control peptide and control protein (cytochrome P450IID6, Pharmacia, Milton Keynes, UK) as liquid phase competitors. Antibody detection was carried out under identical conditions to those described in *ELISA*.

*Statistical analysis*

Results are presented as mean ± SD or as percentages (%). Data were analysed using *t*-test, Mann-Whitney U-test, chi-square ($\chi^2$) test and the Fisher's exact test as appropriate. Correlations between variables were assessed using the Spearman rank order correlation coefficient. A two-tailed $p$ value less than 0.05 was considered significant. Statistical analyses were performed using SPSS (SPSS Inc., Chicago, IL, USA) statistical package.

### Results

*Protein database search and analysis*

Amino acid similarities between SHBsAg (226 aa) and the MPB (304 aa) and MOG (247 aa) antigens are illustrated in Figure 1. Overlapping sequences within $MOG_{10-22}$ share a 66–86% local homology with $SHBsAg_{135-140}$ and $SHBsAg_{83-98}$. Another pair of viral/self mimics involves $SHBsAg_{9-15}$ and $MOG_{218-224}$ (100% homology). $SHBsAg_{135-140}$ is part of the immunodominant "a" B-cell epitope on SHBsAg; $SHBsAg_{83-98}$ is part of the leucine zipper pattern of SHBsAg and $MOG_{10-22}$ extensively overlaps with the encephalitogenic epitope $MOG_{1-20}$ (Landschulz et al. 1988; Genain et al. 1999).

The best homology between SHBsAg and MBP is that of the $SHBsAg_{67-80}$/$MBP_{82-95}$ pair (8/14 homology) (Figure 1).

### ELISA

*Before vaccination*

Reactivity to at least one of the SHBsAg peptides was found in 8 (14%) pre-vaccinated subjects (including 3 cases with antibodies to the full-length SHBsAg) who were excluded from the study.

Amongst the remaining 50 cases, reactivity to at least one of the MOG peptides was present in 4 (8%) (Table I).

*At 3 months post-vaccination*

Reactivity to at least one of the SHBsAg peptides was present in 41/45 (91%) of the vaccinated subjects (Table I), all of whom reacted with $SHBsAg_{130-149}$ (Table II). Antibodies against the full-length SHBsAg were present in 40/45 (89%) of the cases.

Reactivity to at least one of the MOG mimics was present in 24/45 (53%) of the cases, all but one reacting with $MOG_{5-24}$ (Table I and II).

Double reactivity to at least one SHBsAg/MOG mimicking pairs was present in 24/45 (53%) of the cases (Table I and II).

*At 6 months post-vaccination*

Reactivity to at least one of the SHBsAg peptides was present in 36/39 (92%) of the cases (Table I), all of who reacted with $SHBsAg_{130-149}$. All 36 cases also had antibodies against the full-length SHBsAg.

Overall, 47/50 (94%) of the post vaccinated cases had anti-SHBsAg antibody protective immunity (median anti-SHBsAg antibody titre: 386 mIU/ml, range 16–1000 mIU/ml, cut off = 10 mIU/ml).

Reactivity to at least one of the MOG mimics was present in 17/39 (44%) of the cases. At 6 months post-vaccination, 3 of the 4 anti-MOG reactive cases before vaccination and 7 of the 24 (29%) of the anti-MOG reactive cases at 3 months post-vaccination had lost their reactivity to $MOG_{5-24}$.

Double reactivity to at least one SHBsAg/MOG mimicking pairs was present in 16/39 (41%) of the cases.

Overall 30/50 (60%) vaccinees had SHBsAg/MOG double reactivity on at least one occasion (either at 3 or 6 months post-vaccination) compared to none before-vaccination ($p < 0.001$) and in 2/87 (2%, $p < 0.001$) of the pathological controls (a 52-year old woman with PBC and a 28-year old woman with SLE). None of the vaccinees or the pathological controls had double reactivity to the SHBsAg/MBP mimicking pair.

Table II. Antibody reactivity to $SHBsAg_{130-149}$ and $MOG_{5-24}$ before and after vaccination.

| Case # | $SHBsAg_{130-149}$ pre-HB Vacc baseline | $MOG_{5-24}$ pre-Hbvacc baseline | $SHBsAg_{130-149}$ post-HB Vacc 3 months | $MOG_{5-24}$ post-HB vacc 3 month | $SHBsAg_{130-149}$ post-HBvacc 6 months | $MOG_{5-24}$ post-HBVacc 6 months |
|---|---|---|---|---|---|---|
| 1 | 0 | 3.7 | na | na | 7.1 | 0 |
| 2 | 0 | 0 | 4.7 | 3.3 | 5.5 | 0 |
| 3 | 0 | 0 | 3.4 | 0 | na | na |
| 4 | 0 | 0 | 6.7 | 0 | 6.9 | 0 |
| 5 | 0 | 0 | 3.8 | 3.3 | 4.2 | 0 |
| 6 | 0 | 0 | 4.2 | 4 | 6 | 4.8 |
| 7 | 0 | 0 | 0 | 0 | 0 | 0 |
| 8 | 0 | 4.1 | 7.1 | 5.2 | 7.8 | 0 |
| 9 | 0 | 0 | 6.8 | 0 | 5.6 | 0 |
| 10 | 0 | 0 | 6.6 | 2.3 | na | na |
| 11 | 0 | 0 | na | na | 6.5 | 5 |
| 12 | 0 | 0 | 4.3 | 0 | 5.7 | 3.7 |
| 13 | 0 | 0 | 2.8 | 2.8 | 3.9 | 3.9 |
| 14 | 0 | 0 | 2.3 | 0 | 2.7 | 0 |
| 15 | 0 | 0 | na | na | 8.1 | 0 |
| 16 | 0 | 0 | 0 | 0 | 0 | 0 |
| 17 | 0 | 0 | 3.3 | 0 | 6.8 | 0 |
| 18 | 0 | 0 | 3.3 | 4 | 4.9 | 0 |
| 19 | 0 | 0 | 2.8 | 0 | 2.9 | 0 |
| 20 | 0 | 3.2 | 4.1 | 2.9 | 4.1 | 0 |
| 21 | 0 | 0 | 4.7 | 0 | na | na |
| 22 | 0 | 0 | 4.8 | 0 | na | na |
| 23 | 0 | 0 | 3.2 | 2.3 | 3.1 | 3.8 |
| 24 | 0 | 0 | 0 | 0 | 0 | 0 |
| 25 | 0 | 0 | 3.2 | 0 | 3.1 | 4.4 |
| 26 | 0 | 0 | na | na | 7.8 | 3 |
| 27 | 0 | 0 | 2.9 | 0 | 2.9 | 0 |
| 28 | 0 | 0 | 3 | 0 | na | na |
| 29 | 0 | 0 | 3 | 3.2 | na | na |
| 30 | 0 | 0 | 3.1 | 0 | 3 | 0 |
| 31 | 0 | 0 | 5.1 | 3 | 5.1 | 2.2 |
| 32 | 0 | 0 | 3.8 | 3.7 | 6.5 | 3.5 |
| 33 | 0 | 0 | 3.3 | 3 | 4.8 | 4.8 |
| 34 | 0 | 0 | 2.9 | 2.7 | na | na |
| 35 | 0 | 0 | 5 | 5.9 | 5 | 0 |
| 36 | 0 | 0 | 6.1 | 5 | na | na |
| 37 | 0 | 0 | 3.4 | 3 | 3.4 | 3 |
| 38 | 0 | 0 | na | na | 4.8 | 2.9 |
| 39 | 0 | 4.2 | 5.2 | 0 | 5.9 | 0 |
| 40 | 0 | 0 | 3.5 | 3 | 3.5 | 2.6 |
| 41 | 0 | 0 | 2.9 | 2.4 | 2.9 | 2.9 |
| 42 | 0 | 0 | 7 | 0 | 7 | 0 |
| 43 | 0 | 0 | 3.3 | 0 | 3.3 | 3.1 |
| 44 | 0 | 0 | 2.9 | 2.1 | 7.8 | 2.9 |

Table II – *continued*

| Case # | $SHBsAg_{130-149}$ pre-HB Vacc baseline | $MOG_{5-24}$ pre-Hbvacc baseline | $SHBsAg_{130-149}$ post-HB Vacc 3 months | $MOG_{5-24}$ post-HB vacc 3 month | $SHBsAg_{130-149}$ post-HBvacc 6 months | $MOG_{5-24}$ post-HBVacc 6 months |
|---|---|---|---|---|---|---|
| 45 | 0 | 0 | 0 | 0 | 3.8 | 3.1 |
| 46 | 0 | 0 | 5.1 | 4 | na | na |
| 47 | 0 | 0 | 3.3 | 2.2 | 3.2 | 0 |
| 48 | 0 | 0 | 3.8 | 0 | na | na |
| 49 | 0 | 0 | 4.6 | 3.5 | na | na |
| 50 | 0 | 0 | 4.9 | 0 | 4.7 | 0 |

HBVacc, hepatitis B vaccination; SHBsAg, small hepatitis B surface antigen; MOG, myelin oligodendrocyte glycoprotein; na, not available.

None of the vaccinees reported symptoms of demyelinating disorders at the 6-month post-vaccination follow-up.

*Inhibition studies*

Antibody binding to $SHBsAg_{130-149}$ was inhibited by 73–92% after pre-incubation with the $SHBsAg_{130-149}$; by 69–85% after pre-incubation with $MOG_{5-24}$ and by 74–88% after pre-incubation with full-length SHBsAg, in all 3 cases tested (Figure 2). Insignificant inhibition was observed by pre-incubation with the irrelevant control peptide and antigen (Figure 2).

### Discussion

In the present study we have looked for evidence of cross-reactivity between SHBsAg and myelin mimics in normal subjects undergoing hepatitis B vaccination. Not only does SHBsAg share strong homologies with major myelin antigens such as MBP and MOG but also specific viral/self pairs were found to be targets of antibody responses induced by the administration of the viral vaccine. As for other previously described mimics (Quaratino et al. 1995; Bogdanos et al. 2004a, b, 2005), sequence similarity *per se* did not necessarily lead to anti-peptide reactivity and immunological cross-reactivity (Oldstone 1987). In the present study the SHBsAg/MBP mimicking sequences and two of four sets of SHBsAg/MOG mimics were not antibody targets (Table I). Inhibition studies have demonstrated the cross-reactive nature of the observed SHBsAg/-MOG antibody responses (Figure 2). The selective appearance of viral/myelin cross-reactive responses in some but not all of the mimicking pairs supports the biological significance of the present findings (Oldstone, 1987, 1989, 1998).

The question of a connection between vaccination and autoimmune disorders is surrounded by controversy (Herroelen et al. 1991; Cohen and Shoenfeld 1996; Maillefert et al. 1999; Shoenfeld and Aron-Maor, 2000; Aron-Maor and Shoenfeld, 2001; Vital et al. 2002; Poirriez 2004; Ravel et al. 2004; Girard 2005; Selmi et al. 2005). A heated debate is going on regarding the causality between anti-HBV vaccination and demyelinating disorders such as multiple sclerosis (Cohen and Shoenfeld 1996; Marshall 1998; Aron-Maor and Shoenfeld 2001; Ascherio et al. 2001; Confavreux et al. 2001; Geier and Geier 2001; Girard 2005; Selmi et al. 2005). The focus of the present study was the application of HBV vaccination as a model for the study of immunological cross-reactivity rather than the study of the safety and efficacy of the anti-HBV vaccination. Serum samples from HBV vaccination recipients with established autoimmune disorders have not been tested in the present study. All of the vaccinees were free of autoimmune phenomena before vaccination and remain free of any adverse

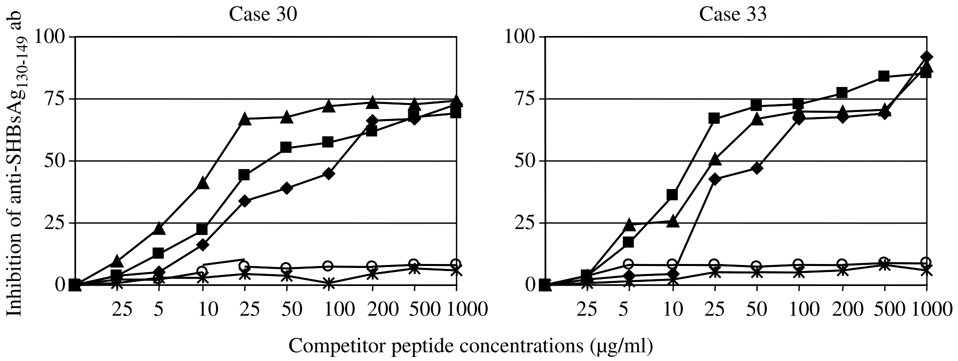

Figure 2. Inhibition of antibody binding against $SHBsAg_{130-149}$ by pre-incubation with $SHBsAg_{130-149}$ (◆), $MOG_{5-24}$ (■), SHBsAg (▲), control antigen (○) and control peptide (*) of case 30 and 33. On the vertical axis is the percentage of inhibition of antibody binding to the peptide in the presence of competitor peptide or antigen at different concentrations. $SHBsAg_{130-149}$, Small Hepatitis B surface antigen; MOG, myelin oligodendrocyte glycoprotein; ab, antibody reactions during the follow up. Clues as to the pathogenic link between the vaccine and specific autoimmune disorders, therefore, could not be established. Cross-reactive immunity was found only after vaccination, though decreasing in magnitude over time with a significant proportion of vaccinated subjects maintaining the anti-viral response but losing that against self. These findings suggest that upon vaccination, induction of an anti-viral response is initially capable of promoting cross-reactive anti-self immune responses, which decrease over time, possibly as a result of peripheral tolerance mechanisms. This scenario may explain why very rarely adverse post-vaccination autoimmune reactions occur (Ascherio et al. 2001; Confavreux et al. 2001). Equally important may be the finding that the minority of vaccinees with an anti-myelin reactivity pre-HBV vaccination almost universally lost this reactivity post-vaccination. It is possible to speculate that the viral components of the vaccine and in particular the self mimicking SHBsAg sequences may play a role as altered peptide ligand, i.e. may represent sequences unable to induce cross-reactive responses but able to promote tolerance to a given autoepitope (Sloan-Lancaster and Allen 1996). Such mechanism could probably be of benefit in patients with multiple sclerosis in whom HBV vaccination or immunomodulatory treatment with SHBsAg mimics as altered peptide ligand might contribute to restoration of tolerance towards myeling antigens (Sloan-Lancaster and Allen 1996).

The hepatitis B vaccine is the first vaccine which has been shown to prevent cancer, and it has saved—and will save—millions of lives (Shouval 2003). But is also a vaccine against a virus which has been pathogenetically linked to autoimmune adverse reactions (Herroelen et al. 1991; Cohen and Shoenfeld 1996; Maillefert et al. 1999; Shoenfeld and Aron-Maor 2000; Aron-Maor and Shoenfeld 2001; Vital et al. 2002; Poirriez 2004; Ravel et al. 2004). In view of the observed SHBsAg/MOG cross-reactivity, the vaccine's possible role as a trigger for the induction and/or maintenance of viral/self cross-reactivity through molecular mimicry must be further investigated.

### Acknowledgements

This project was supported by an Innovative Award of the Multiple Sclerosis Society, London, UK. DP Bogdanos, and G Mieli-Vergani are supported by the Children's Liver Disease Foundation (Birmingham, UK). G Mieli-Vergani is also supported by WellChild (Cheltenham, UK).

### References

Aron-Maor A, Shoenfeld Y. 2001. Vaccination and systemic lupus erythematosus: The bidirectional dilemmas. Lupus 10:237–240.

Ascherio A, Zhang SM, Hernan MA, Olek MJ, Coplan PM, Brodovicz K, Walker AM. 2001. Hepatitis B vaccination and the risk of multiple sclerosis. N Engl J Med 344:327–332.

Bogdanos DP, Mieli-Vergani G, Vergani D. 2000. Virus, liver and autoimmunity. Dig Liver Dis 32:440–446.

Bogdanos DP, Choudhuri K, Vergani D. 2001. Molecular mimicry and autoimmune liver disease: Virtuous intentions, malign consequences. Liver 21:225–232.

Bogdanos DP, Baum H, Grasso A, Okamoto M, Butler P, Ma Y, Rigopoulou E, Bogdanos DP, Baum H, Sharma UC, Grasso A, Ma Y, Burroughs AK, Vergani D. 2002. Antibodies against homologous microbial caseinolytic proteases P characterise primary biliary cirrhosis. J Hepatol 36:14–21.

Bogdanos DP, Baum H, Grasso A, Okamoto M, Butler P, Ma Y, Rigopoulou E, Montalto P, Davies ET, Burroughs AK, Vergani D. 2004a. Microbial mimics are major targets of crossreactivity with human pyruvate dehydrogenase in primary biliary cirrhosis. J Hepatol 40: 31–9.

Bogdanos DP, Baum H, Gunsar F, Arioli D, Polymeros D, Ma Y, Burroughs AK, Vergani D. 2004b. Extensive homology between the major immunodominant mitochondrial antigen in primary biliary cirrhosis and Helicobacter pylori does not lead to immunological cross-reactivity. Scand J Gastroenterol 39: 981–987.

Bogdanos DP, Baum H, Okamoto M, Montalto P, Sharma UC, Rigopoulou EI, Vlachogiannakos J, Ma Y, Burroughs AK, Vergani D. 2005. Primary biliary cirrhosis is characterized by IgG3 antibodies cross-reactive with the major mitochondrial autoepitope and its lactobacillus mimic. Hepatology 42:458–465.
Cohen AD, Shoenfeld Y. 1996. Vaccine-induced autoimmunity. J Autoimmun 9:699–703.
Confavreux C, Suissa S, Saddier P, Bourdes V, Vukusic S. 2001. Vaccinations and the risk of relapse in multiple sclerosis. Vaccines in multiple sclerosis study group. N Engl J Med 344:319–326.
Devaux B, Enderlin F, Wallner B, Smilek DE. 1997. Induction of EAE in mice with recombinant human MOG, and treatment of EAE with a MOG peptide. J Neuroimmunol 75:169–173.
Dyrberg T, Oldstone MB. 1986. Peptides as probes to study molecular mimicry and virus-induced autoimmunity. Curr Top Microbiol Immunol 130:25–37.
Geier MR, Geier DA. 2001. Immunologic reactions and hepatitis B vaccine. Ann Intern Med 134:1155.
Genain CP, Cannella B, Hauser SL, Raine CS. 1999. Identification of autoantibodies associated with myelin damage in multiple sclerosis. Nat Med 5:170–175.
Girard M. 2005. Autoimmune hazards of hepatitis B vaccine. Autoimmun Rev 4:96–100.
Herroelen L, de Keyser J, Ebinger G. 1991. Central-nervous-system demyelination after immunisation with recombinant hepatitis B vaccine. Lancet 338:1174–1175.
Kerkar N, Choudhuri K, Ma Y, Mahmoud A, Bogdanos DP, Muratori L, Bianchi F, Williams R, Mieli-Vergani G, Vergani D. 2003. Cytochrome P4502D6(193-212): A new immunodominant epitope and target of virus/self cross-reactivity in liver kidney microsomal autoantibody type 1-positive liver disease. J Immunol 170:1481–1489.
Landschulz WH, Johnson PF, McKnight SL. 1988. The leucine zipper: A hypothetical structure common to a new class of DNA binding proteins. Science 240:1759–1764.
Maillefert JF, Sibilia J, Toussirot E, Vignon E, Eschard JP, Lorcerie B, Juvin R, Parchin-Geneste N, Piroth C, Wendling D, Kuntz JL, Tavernier C, Gaudin P. 1999. Rheumatic disorders developed after hepatitis B vaccination. Rheumatology (Oxford) 38: 978–983.
Marshall E. 1998. A shadow falls on hepatitis B vaccination effort. Science 281:630–631.
Martin R, McFarland HF, McFarlin DE. 1992. Immunological aspects of demyelinating diseases. Annu Rev Immunol 10:153–187.
Montalto P, Davies ET, Burroughs AK, Vergani D. 2004a. Microbial mimics are major targets of crossreactivity with human pyruvate dehydrogenase in primary biliary cirrhosis. J Hepatol 40:31–39.
Oldstone MB. 1989. Molecular mimicry as a mechanism for the cause and a probe uncovering etiologic agent(s) of autoimmune disease. Curr Top Microbiol Immunol 145:127–135.
Oldstone MB. 1998. Molecular mimicry and immune-mediated diseases. Faseb J 12:1255–1265.
Oldstone MB. 1987. Molecular mimicry and autoimmune disease. Cell 50:819–820.
Poirriez J. 2004. A preliminary experiment of absorption of antinuclear antibodies by the hepatitis B vaccine components, in a case of neurolupus. Vaccine 22:3166–3168.
Quaratino S, Thorpe CJ, Travers PJ, Londei M. 1995. Similar antigenic surfaces, rather than sequence homology, dictate T-cell epitope molecular mimicry. Proc Natl Acad Sci USA 92:10398–10402.
Raine CS, Bornstein MB. 1970. Experimental allergic encephalomyelitis: a light and electron microscope study of remyelination and "sclerosis" *in vitro*. J Neuropathol Exp Neurol 29:552–574.
Ravel G, Christ M, Horand F, Descotes J. 2004. Autoimmunity, environmental exposure and vaccination: Is there a link? Toxicology 196:211–216.
Selmi C, Battezzati PM, Gershwin ME, Tishler M, Shoenfeld Y. 2005. Vaccines in the 21st century: The genetic response and the innocent bystander. Autoimmun Rev 4:79–81.
Shoenfeld Y, Aron-Maor A. 2000. Vaccination and autoimmunity-'vaccinosis': A dangerous liaison? J Autoimmun 14:1–10.
Shouval D. 2003. Hepatitis B vaccines. J Hepatol 39(Suppl. 1): S70–S76.
Sloan-Lancaster J, Allen PM. 1996. Altered peptide ligand-induced partial T cell activation: molecular mechanisms and role in T cell biology. Annu Rev Immunol 14:1–27.
Steinman L. 1996. Multiple sclerosis: A coordinated immunological attack against myelin in the central nervous system. Cell 85:299–302.
Van de Water J, Ishibashi H, Coppel RL, Gershwin ME. 2001. Molecular mimicry and primary biliary cirrhosis: Premises not promises. Hepatology 33:771–775.
Vital C, Vital A, Gbikpi-Benissan G, Longy-Boursier M, Climas MT, Castaing Y, Canron MH, Le Bras M, Petry K. 2002. Postvaccinal inflammatory neuropathy: Peripheral nerve biopsy in 3 cases. J Peripher Nerv Syst 7:163–167.
Wekerle H. 1999. Remembering MOG: autoantibody mediated demyelination in multiple sclerosis? Nat Med 5:153–154.
Wucherpfennig KW. 2001. Mechanisms for the induction of autoimmunity by infectious agents. J Clin Invest 108: 1097–1104.
von Herrath MG, Fujinami RS, Whitton JL. 2003. Microorganisms and autoimmunity: Making the barren field fertile? Nat Rev Microbiol 1:151–157.

